# Quality of life in overweight and obese young Chinese children: a mixed-method study

**DOI:** 10.1186/1477-7525-11-33

**Published:** 2013-03-06

**Authors:** Christine Mei Sheung Chan, Wen-Chung Wang

**Affiliations:** 1 Department of Psychological Studies, The Hong Kong Institute of Education, Hong Kong, Hong Kong

**Keywords:** Childhood obesity, Quality of life, Socio-demographics, Chinese young children and parents, Mixed method study

## Abstract

**Background:**

Obesity among young children in Hong Kong has become a public health problem. This study explored associations between Chinese parent reported children’s quality of life (QoL), socio-demographics and young children’s weight status from 27 preschool settings.

**Methods:**

A mixed-method approach, including quantitative and qualitative tools, was employed for this cross-sectional study. Quantitative data were collected from 336 Chinese parents of children aged 2–7 years. Paediatric Quality of Life Inventory 4.0 (PedsQL, v 4.0) and a questionnaire about parents’ socio-demographics were used. In-depth interviews with mothers, teachers and children from a larger sample were the basis of 10 case studies. Quantitative data were analysed using chi-square analysis, one-way ANOVA and logistic regression. Qualitative data were analysed according to a multi-level framework that established linkages with quantitative data.

**Results:**

The children’s Body Mass Index (BMI) ranged from 11.3 to 28.0 kg/m^2^ and was classified into four weight groups. ANOVAs showed that the normal-weight children had significantly higher PedsQL scores in Physical Functioning than obese children (mean difference = 14.19, *p* < .0083) and significantly higher scores in School Functioning than overweight children (mean difference = 10.15, *p* < .0083). Results of logistic regression showed that relative to normal-weight children, obese children had a 2–5 times higher odds of showing problems in Physical, Social Functioning and School Performance. Overweight children had 2 times higher odds of problems in Social Functioning, and underweight children had a 2 times higher odds of problems in Physical Functioning. Children’s age (*χ*^2^ = 21.71, *df* = 3, *p* < 0.01), and housing (*χ*^2^ = 33.00, *df* = 9, *p* < 0.01) were associated with their weight. The case studies further act as a supplement to the quantitative data that children showed emotional problems across different abnormal weight statues; and the association between children’s weight status and well-being might be affected by multiple childcare arrangements and familial immigration status.

**Conclusions:**

This study is one of only a few studies that have examined parents’, teachers’ and young children’s own perceptions of the children’s quality of life across different weight statuses. The results are discussed in terms of their implications for intervention.

## Introduction

Studies have shown that there is a consistent relationship between abnormal weight and the perception of low quality of life in children [1,2]. Quality of life is defined by Felce and Perry [3] as overall well-being described by objective indicators and subjective evaluation of physical, material, social, productive, emotional and civic well-being. It can be seen as a multidimensional construct that reflects one’s self-perceptions of enjoyment, satisfaction with life [4] and general health [5].

There is growing awareness that overweight status is associated with significant physical and psychosocial health problems in adolescents and is a precursor for adult obesity and disease. The most widespread correlates of early childhood obesity may also be psychosocial, including lower perceived competency than seen in normative samples in the social, physical, and appearance domains, as well as lower perceived overall self-worth [6]. These areas are essential for healthy child development. Though a strong research body has suggested that quality of life should be evaluated among overweight/obese children to identify effective weight intervention and management strategies [7,8], medical staff and health workers often do not offer treatment to young, obese children in the absence of co-morbid conditions [9].

Childhood obesity has reached epidemic proportions and has become a major global public health problem [10]. In Hong Kong, between 1988 and 2008, the prevalence of obesity almost tripled among children in primary grades, and it is predicted that one-fourth of children will be obese by 2013 [11]. The evidence suggests that obese young children currently aged 4.5 years old are being more likely 5 times to develop obesity by the time they are 12 years old [12]. Approximately 55,318 (17%) of children under 5 years of age were overweight in 2010, as reported by the Hong Kong Government [13].

Socio-demographic issues may also be important in explaining the increase in childhood obesity in Hong Kong, which has experienced social and political changes since becoming one of the Special Administrative Regions of the People’s Republic of China in 1997. According to official government reports, many children suffer from higher rates of parental separation, divorce, domestic violence, unemployment, suicidal tendencies and labour mobility than were seen in the past [14-16]. Children’s weight problems in Hong Kong could also be influenced by increasing numbers of young families migrating from mainland China [17], where childhood obesity rates are also high. Between 1989 and 1997, the prevalence of obesity in mainland China increased from 1.5% to 12.6% in urban areas, with a reported prevalence of 11-18% in cities and 3 - 4% in rural areas [18]. Again, little is known about the weight status and quality of life of these young children living in Hong Kong.

In response to the above issues, we hypothesise, with a view to inform education and health policy makers, that there may be a direct relationship between child weight and quality of life; and there may be an indirect relationship between child weight and quality of life through the effects of the socio-demographics of parents. The specific research questions are as follows:

1. To what extent is there a relationship between children’s weight and parents’ socio-demographic characteristics?

2. To what extent is there a relationship between the quality of life and weight status of young children?

3. To what extent can quality of life outcomes be explained across case studies by young children’s weights?

## Methods

A mixed-method approach, including quantitative and qualitative tools, was employed for this cross-sectional study. Mixed-method techniques were used to expand the scope of the study and to deepen the insights into the subjects’ perceptions of young children’s weight [19-21]. The purpose of using both quantitative and qualitative methods is so that each method will complement the other’s strengths and weaknesses and will contribute to a more complete understanding of the research questions [22,23]. Case studies typically examine the interplay among variables that the quantitative findings identified as important, to provide a complete understanding of a contemporary phenomenon within its real-life context [24].

### Instrument

The PedsQL™ version 4.0 [25] was selected to measure children’s health-related quality of life, and was completed by the children’s parents. The questionnaire yielded a total score (Total PedsQL) and scores on 4 separate scales that were used to assess the children’s level of difficulty with Physical Functioning (PF-8 items), Emotional Functioning (EF-5 items), Social Functioning (SF-5 items) and School Functioning (SchF-5 items). Psychosocial Summary Health was a composite of the Emotional, Social, and School Functioning Scales. Each subscale was based on a 5-point Likert scale. Items were scored as 0, 25, 50, 75 and 100. We calculated scale scores by dividing the sum of the item scores by the number of items. All scores ranged from 0 to 100, with a higher score indicating better quality of life. The existing Chinese language version of the PedsQL shows satisfactory validity and reliability [26]. In the present study, Cronbach’s alpha ranged from 0.67 - 0.91 for children aged 2–4 years and from 0.74 - 0.90 for children aged 5–7 years. We only used the mother-report of the PedsQL for the children aged 2–7 years in the quantitative study.

Questionnaires concerning the parents’ socio-demographic characteristics included questions about being perceived as the primary child caregiver, birth place, length of stay in Hong Kong, educational levels, types of housing and employment status; questions about the children included gender, number of siblings, types of schooling, number of stages of childcare arrangements since birth. The SES of the individual families was also defined by parental education, housing and family monthly income.

The protocol for the face to face semi-structured for the case studies was based on the PedsQL questionnaire, that the mothers and teachers had completed, and children who were interviewed completed a self-report version of the PedsQL (see Additional file 1) if they were age 5 or older. Interviewers obtained examples of the children’s quality of life and asked respondents’ about their reasons for answering the questionnaire items as they did.

### Definitions and weight measurement of young Chinese children

The children’s heights and weights were routinely measured by their kindergarten teachers as part of the usual curriculum. The International Obesity Task Force (IOTF) was used to determine the children’s weight status. The IOTF’s weight categories were based on age-and sex-based norms, so that children with the same BMI might be classified differently based on age or sex. According to the IOTF’s weight categories, obesity and overweight are defined according to international BMI cut-off points by sex for exact age between 2–18 years of age, with a BMI of 25 and 30 kg/m^2^ at age 18 defined as being overweight and obese, respectively [27]; underweight is defined according to the international BMI cut-off points by sex for exact ages between 2–18 years of age, and includes a BMI cut-off of 16 kg/m^2^, which was obtained by averaging data from Brazil, Great Britain, Hong Kong, Netherlands, Singapore and the United States [28]. Since the development of the IOTF standards included the Hong Kong population, it has been proposed by the local government using this tool to measure Hong Kong children’s weight status [29]. According to the IOTF definition, child BMI is classified into 4 mutually exclusive categories: underweight, normal weight, overweight and obese.

### Participants

Three hundred and thirty-six parents of children aged 2–7 years from 27 kindergartens and childcare centres of different socio-economic status (SES) in the city of Hong Kong participated in the study. In order to have varying levels of SES represented in the sample, a range of kindergartens were selected based on geographical location (ranging from private housing areas to public housing areas) and school fees. One hundred and sixty parents had a child aged 5–7 years and 176 parents had a child aged 2–4 years. Parental consent forms were obtained by the participating kindergartens. The 10 case studies were recruited voluntarily from the sample pool. Two children were underweight, three were normal weight, two were overweight and two were obese. We deliberately sought parents who actively looked after their children and children of different weight status from different kindergartens; we then approached teachers and children individually to provide information for the case studies. Ethical approval was obtained from the Research and Development Office at the Hong Kong Institute of Education.

### Data collection

To provide assistance to individuals with limited literacy, the questionnaire was distributed in a group setting, typically including 4 to 6 people. First, the parents were invited to complete the demographic sheet with assistance. Second, the entire group of parents completed the PedsQL questionnaire. For parents who had difficulty understanding the questionnaire, individual assistance was offered. For the case studies, mothers and teachers were interviewed face to face, and the self- report questionnaire data were collected from children aged 5–7 individually, one question at a time. Small gifts were given to the participating children. All interviews which were tape-recorded were first transcribed in the local Chinese dialect (Cantonese) and then backward translation was also conducted to ensure the quality of translation.

### Statistical analysis

There were several stages of the statistical analysis. First, Chi-square tests were used to explore the association between demographic variables and child weight status. Fisher’s exact tests were used to explore the association between the remaining demographic variables (perceived as principal caregiver and father’s employment status) and child weight status. Second, a correspondence analysis was used to display the patterns of SES variables having a significant association with weight status [30]. Third, descriptive statistics provided the means and standard deviations of the dimensional scores on the PedsQL, including PF, EF, SF, SchF, and Psychosocial Health Summary scores, across different weight groups. Univariate analyses of variance (one-way ANOVAs) were employed to determine whether there were any differences among the group means of the PedsQL subscale scores. Tukey’s Honestly Significant Difference (Tukey’s HSD) post-hoc test was used to calculate the pair-wise *p*-values of different groups on the PedsQL subscales because the confidence interval of the Tukey’s HSD is narrow (i.e., the test is more powerful) than that of other commonly used methods [31]. Effect sizes, in the form of eta-squared, were also computed to examine practical significance, as statistical significance would be severely influenced by a large sample size. Eta-squared is equal to the between-group sum-of-squares divided by the total sum-of-squares. In this case, eta-squared represents the proportion of variance in the PedsQL subscale scores (dependent variables) explained by children’s weight status.

Independent samples *t*-tests were used to test differences in PedsQL subscale scores between demographic groups. Finally, the PedsQL scores were dichotomised as above or below the bottom quartile, for each subscale. Multiple logistic regression analyses were used to compute odds ratios describing the odds of falling into the bottom quartile on the PedsQL subscales, given the child’s weight status. Covariates included in each multiple logistic regression analysis were selected using a logistic regression with backward elimination, retaining variables that were significant at *p* < .10.

PedsQL subscale scores act as dependent variables and weight status of children act as independent variables in the logistic regression. Different demographic factors had effects on different PedsQL subscales; therefore, we used different covariates in different PedsQL subscales analyses. The reference group for all logistic analyses was the normal weight children, with the other categories coded as “dummy variables.”

To test for normality in the distribution of error scores we examined the Q-Q plots of the residuals of the subscales, and found that all were normally distributed. However, by the Levene’s test, we found that scores on the Physical Functioning subscale violated the assumption of homogeneity of variance (*F* (3,332) = 7.61, *p* < 0.01). Therefore to determine the effect of weight on PedsQL subscales we used Welch ANOVA for Physical Functioning and ANOVA for the other subscales. Tukey’s HSD and Tamhane’s T2 post-hoc tests were used to make pair-wise comparisons of group means on the PedsQL subscales. Tamhane's T2 is a conservative test and is considered more appropriate than Tukey's HSD when cell sizes are unequal, or when homogeneity of variances is violated. Bonferroni correction was used to counteract the problem of multiple comparisons. It is considered the simplest and most conservative method to control the family wise error rate. Therefore, p < 0.0083 was used for pair-wise comparisons.

The possibility of confounds is an important consideration. Although we were not able to examine a wide range of possible confounds, we were able to check on the possibility that quality of life scores differed based on relevant demographic characteristics. Although the independent sample t-tests did show significant differences in Physical Functioning between the two age groups, the Pearson’s correlation showed only a weak association between physical functioning and age (*r* = −0.227) [32]. No other demographic variables were significantly associated with the quality of life scores. As a result, we do not believe that the demographic characteristics we examined had a confounding effect in the current study.

Finally, the backward transcripts from the taped interviews were analysed according to a multi-level framework that established linkages between the children’s four quality of life domains and their functioning in daily life. The interviews were brief enough that we could analyse them as a “whole” classified into four groups of weight statues. We then incorporated data from the quantitative findings to make overall conclusions.

## Results

### To what extent is there a relationship between child weight and socio-demographics?

#### Child characteristics

Of the 336 children in the study, 52.3% were aged 2–4 years and 47.6% were aged 5–7 years; 53.5% of the children were boys, and 46.4% were girls. According to the classification of the IOTF, 72% of children had abnormal weights and 28% had normal weights; 94 children were underweight (BMI 11.3-14.7), 93 children were overweight (BMI 17.2-19.5); 55 children were obese (BMI 19.2-28.0); and 94 children were normal weight (BMI 14.1-17.5). All children in the study had one or more siblings; however, overweight (65.6%), obese (56.4%) and underweight children were more likely to be an only child than normal weight children (57.4%). Over half of the underweight (51.1%), overweight (53.8%) and obese children (60%) attended a full-day kindergarten, compared to only 29.8% of the normal weight children. Most children (72.6%) were in their first stage of childcare arrangement since birth, and the remaining children (27.4%) were either in their second or the third stage of childcare arrangement, with multiple caregivers in different places; however, among the four weight categories, underweight children (12.8%) were the group least likely to have had two caregivers. Just over half (56.8%) of the mothers considered themselves the principal caregiver. Other principal caregivers included fathers (2.1%), grandparents, relatives and in-house maids (41.1%).

In short, the majority of young children were either underweight or overweight or obese in this cross-sectional study. The overweight and obese children were more likely to be only children, attending a full-day kindergarten with multiple caregivers. Comparatively, the normal weight children had the opposite profile in this sample.

#### Parent characteristics

Of the 336 parents, 90.2% respondents were mothers and 9.8% were fathers. Among the four child weight status groups, more mothers of underweight (61.7%) and obese children (58.2%) considered themselves principal caregivers than the mothers of normal weight children (55.3%). Regarding details of parents’ place of birth, length of stay in HK, educational background, employment status and other demographics (see Additional file 2). In short, the obese children lived in the most socially disadvantaged housing and were more likely to be from newly arrived immigrant families; most of their mothers who had the shortest stay in Hong Kong were born in China, while their fathers had the longest stays in Hong Kong compared to the other weight groups. However, both parents of normal weight children tended to be born in Hong Kong.

From the Additional file 2, there were statistically significant associations between child weight status and age (χ^2^ = 21.71, *df* = 3, *p* < 0.01), type of schooling (χ^2^ = 17.22, *df* = 3, *p* < 0.01) and housing (χ^2^ = 33.00, *df* = 9, *p* < 0.01). The proportion of children of normal weight aged 2–4 years was higher than the proportion of normal weight children aged 5–7 years (38.6% vs. 16.3%, respectively). However, the proportion of abnormal weight children aged 5–7 years was higher than the proportion of those aged 2–4 years (31.3% vs. 25.0%, respectively, for underweight; 34.4% vs. 21.6%, respectively, for overweight; and 18.1% vs. 14.8%, respectively, for obese). Similarly, the proportion of normal-weight children in full-day kindergarten was higher than the proportion of normal-weight children in half-day kindergarten (37.3% vs. 17.6%, respectively), but the proportion of abnormal-weight children in half-day kindergarten was higher than the proportion of abnormal-weight children in full-day kindergarten (30.2% vs. 26.0%, respectively, for underweight; 31.4% vs. 24.3%, respectively, for overweight; and 20.8% vs. 12.4%, respectively, for obese).

**Figure 1:**
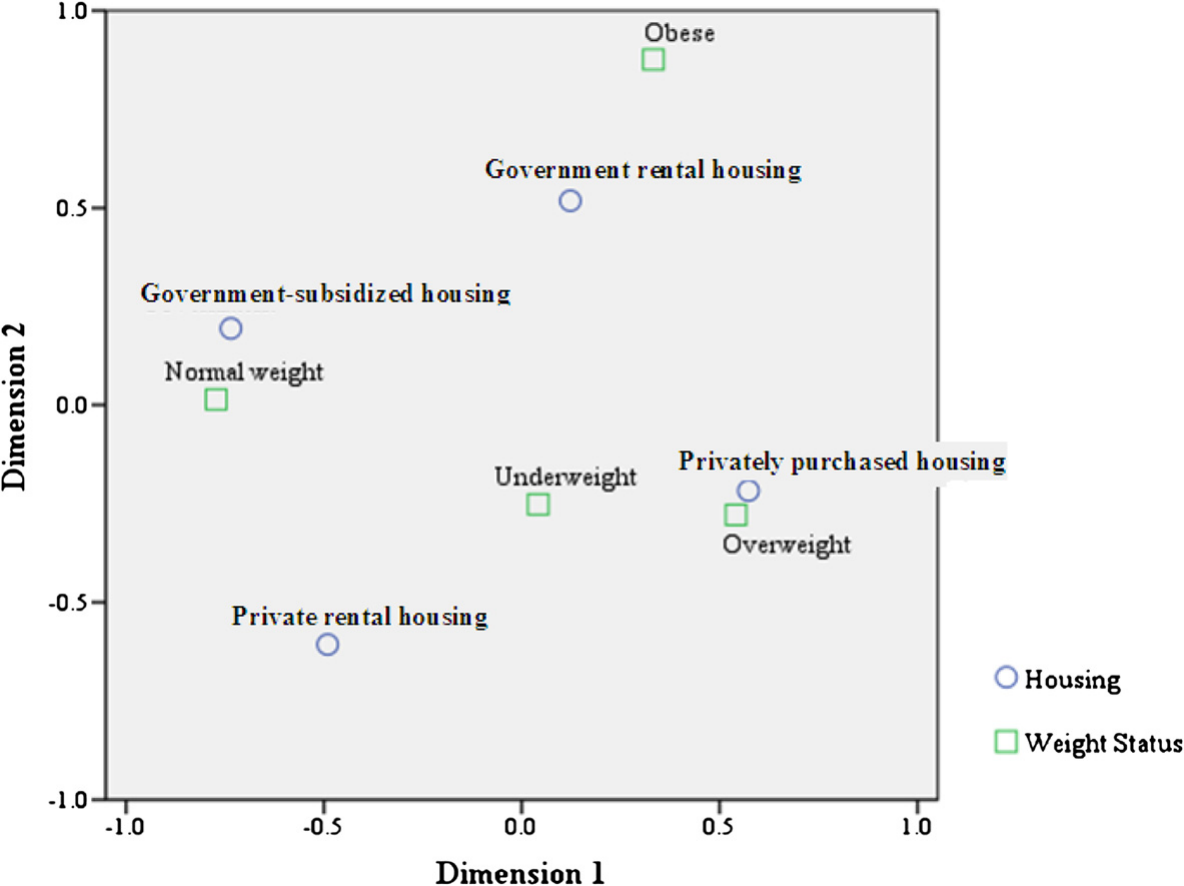
Joint plot of housing and child weight status from correspondent analysis.

From Table 1, it is difficult to render the results in such a way to summarise the structure of a relationship between the multi-level categorical variables for housing type and weight status. Therefore, correspondent analysis was used to help make this structure in the data clear. Figure 1 clearly indicates a relationship between weight status and distance from different housing types; overweight children seems close together in their distances to privately purchased housing; normal-weight children seems to show similar distances to government subsidised housing and obese children were more closely related to government rental housing. However, underweight children were similarly close in distance to the four types of housing. In short, the obese children were most likely indicated as the poorest group among the four groups of children.

**Table 1:** **One-way ANOVA values comparing PedsQL among children with different weight status (*N *= 336)**

**Subscale**	***n***	**Mean**	**SD**	**Difference**	***df***	***F***	***p *value**	**Effect size (η**^**2**^**)**
**PedsQL**								
Physical Functioning				b>d*	3,163	6.100	.001	.0473
Underweight_a_	94	71.14	21.25					
Normal Weight_b_	94	79.42	17.54					
Overweight_c_	93	72.24	23.34					
Obese_d_	55	65.23	24.17					
Emotional Functioning					3,332	.680	.565	.0061
Underweight_a_	94	69.52	16.95					
Normal Weight_b_	94	70.16	17.67					
Overweight_c_	93	72.90	17.56					
Obese_d_	55	70.73	16.14					
Social Functioning					3,332	3.299	.021	.0289
Underweight_a_	94	76.22	16.84					
Normal Weight_b_	94	81.12	16.64					
Overweight_c_	93	76.67	19.62					
Obese_d_	55	71.82	18.09					
School Functioning				b>c*	3,332	5.278	.001	.0455
Underweight_a_	94	67.94	19.46					
Normal Weight_b_	94	75.66	19.33					
Overweight_c_	93	65.50	20.25					
Obese_d_	55	65.09	20.76					
Psychosocial Health Summary					3,332	2.392	.068	.0212
Underweight_a_	94	71.36	13.53					
Normal Weight_b_	94	75.58	15.08					
Overweight_c_	93	71.93	15.59					
Obese_d_	55	69.38	15.01					

*Note*: *p < 0.0083 based on Tukey’s HSD and Tamhane’s T2 post hoc test. Eta squared (η^2^) of 0.0099 indicates a small effect, 0.0588 indicates a medium effect and 0.1379 indicates a large effect [33].

Results of the independent samples *t*-test showed that children aged 2–4 years had significantly higher scores than children aged 5–7 years in Physical Functioning (mean difference = 9.92, *t*_334_ = 4.265, *p* < .01), Social Functioning (mean difference = 5.36, *t*_334_ = 2.754, *p* < .01) and School Functioning (mean difference = 9.11, *t*_334_ = 4.222, *p* < .01). There were no significant differences between the two types of schooling. A one-way ANOVA showed that significant differences were found across types of housing for School Functioning (*F*_*3, 332*_ = 3.01, *p* = 0.030), while the post hoc test demonstrated that no pairs of housing types differed significantly (*p* > 0.0083).

### To what extent is there a relationship between quality of life and the weight status of young children?

Table 1 shows the means and standard deviations for each of the PedsQL subscales by child weight status and the results from the Welch ANOVA for Physical Functioning and ANOVA for other subscales. Significant differences were found in Physical Functioning (*F*_*3, 163*_ = 6.10, *p* = 0.001, effect size η^2^ = .0473), Social Functioning (*F*_*3, 332*_ = 3.30, *p* = 0.021, effect size η^2^ = .0289) and School Functioning (*F*_*3, 332*_ = 5.28, *p* = 0.001, effect size η^2^ = .0455). The findings from the post hoc tests demonstrated that normal-weight children had higher quality of life levels than the abnormal-weight children. For example, the normal-weight children had significantly higher scores in Physical Functioning (mean difference = 14.19, *p* < .0083) than obese children. Moreover, normal-weight children had significantly higher scores in School Functioning (mean difference = 10.15, *p* < .0083) than overweight children.

From Table 2, based on the results of the logistic regression analysis, after adjusting for covariates, obese children had a 5 times higher odds of having a Physical Functioning score in the lowest quartile compared with normal-weight children. Obese children had a 3 times higher odds of having a School Functioning score in the lowest quartile compared with normal-weight children. Obese children also had a 2 times higher odds of having a Social Functioning score in the lowest quartile compared with normal-weight children. Moreover, overweight children had a 2 times higher odds of having a Social Functioning score in the lowest quartile compared with normal-weight children. Underweight children had a 2 times higher odds of having a Physical Functioning score in the lowest quartile compared with normal-weight children. Finally, interestingly, normal-weight children had a 4 times higher odds (1/0.235 = 4.255) of having an Emotional Functioning score in the lowest quartile compared with obese children. The differences for the other subscales according to weight status were not statistically significant.

**Table 2:** Proportions and odds ratios of being in the worst category of pedsQL (the lowest quartile) for obese, overweight and underweight, after adjusting for significant covariates

**Subscale**	**Proportion in the Lowest Score Category, %**	**Odds Ratios (95% CI)**
Physical Functioning§		
Underweight	29.4	2.371 (1.046 – 5.374)
Overweight	29.4	2.269 (0.996 – 5.166)
Obese	28.2	5.308 (2.246 – 12.546)
Emotional Functioning#		
Underweight	26.7	0.679 (0.333 – 1.382)
Overweight	25.3	0.646 (0.310 – 1.347)
Obese	10.7	0.235 (0.074 – 0.743)
Social Functioning#		
Underweight	26.3	1.631 (0.759 – 3.505)
Overweight	29.5	2.160 (1.017 – 4.584)
Obese	24.2	2.450 (1.035 – 5.801)
School Functioning+		
Underweight	24.4	1.333 (0.617 – 2.879)
Overweight	33.7	2.108 (0.996 – 4.459)
Obese	24.4	2.863 (1.272 – 6.445)
Psychosocial Health Summary∥		
Underweight	23.8	1.241 (0.551 – 2.794)
Overweight	30.0	1.989 (0.907 – 4.364)
Obese	22.5	1.935 (0.772 – 4.849)

*Note*:

§Value adjusted for child age, number of childcare arrangements since birth, parents perceived as principal caregiver, mother’s education level and father’s education level.

#Value adjusted for number of stages of childcare arrangement since born, mother’s education level, father’s education level and father’s employment status.

+Value adjusted for child age, housing, mother’s education level, and father’s education level.

∥Value adjusted for mother’s birth place, mother’s length of stay in Hong Kong, mother’s education level, father’s education level and father’s employment status.

### To what extent can quality of life outcomes be explained across case studies by young children’s weights?

Ten parents agreed to be interviewed about their children’s quality of life. These interviews, along with interviews with these ten children, their parents and teachers, were integrated into case study data. For illustrative purposes, we only include four of the case studies here representing the quality of life of one child from each weight category (Case 1: obese; Case 2: overweight; Case 3: underweight; and Case 4: normal weight).

#### Case 1: The obese boy

This boy is 4.5 years old with a BMI of 25.87, placing him in the obese category. The case study interviews showed that he has significant problems with physical, social and emotional functioning, as well as poor school performance. He is the only child in his family and is attending a full-day kindergarten with expensive kindergarten fee. Both parents have tertiary education. He is cared for by his in-house maid together with his grandmother while his parents work full time as a manger in a small shop and an accountant. They live in a private flat.

**The mother said**, “*… he does not like to walk even for half a street…. He normally does not like to run or play actively in the playground nearby.***”***(OM1:13) “*… *he seems to always fight with his friends and often does not like to do his homework…” (OM1:48).****“****I guess he is a happy boy…. Er… I do not know how to reduce his weight, though I notice he is fat now…” (OM2:50). “… He is big but he does not like to eat ice cream…. I need help….I am very busy with my work, like his father…” (OM1:68)*

**The teacher said,***“…. especially he appears not to play around during play time, and he would rather stand in the corner or walk around” (OT2:9). “…. he appears sad and frustrated and is often easily angering by the other children who come near him…. But some children do tease him as a ‘fat kid’” (OT1:27). “… he cannot perform as well as the other children in school… and he can never concentrate in class… he is struggling in his school work” (OT2:64).*

**The boy said**, *“I like to walk and run, but I do not like riding a bicycle… “I feel sad sometimes because my mother is feeling sad…… because I do not play the piano” (OC1:13)…”My friends do not like to play with me. I do not know why… I like to eat ice cream” (OC2:39).*

#### Case 2: The overweight girl

The girl is 5 8/12 years old with a BMI of 18.5, in the overweight category. This case study shows examples of her poor school performance and impaired social and emotional functioning. She is the only child in the family and is attending a full-day kindergarten with average school fees. Both the child and her mother immigrated to Hong Kong from Mainland China two years ago. They live in a rental flat with an in-house maid together with the child’s grandmother and cousin. Both parents have primary education and are working full-time; the father is a shop keeper and the mother works as sales assistant.

**The teacher said,***“…her performance in craft work was very slow… Er… she has poor concentration and easily forgets to do her homework. She is absent from school often due to sickness” (OWT1:45). “… she has difficulty getting along with other children” (OWT1:33).*

**The teacher added**, “…*the girl is quicker to quarrel with people and other children, especially during play activities” (OWT2:56). “… she is unable to play certain activities that the other children play” (OWT1:12).*

**Her mother said,***“… she is usually scared and is afraid of things happening to her…. she quite often has sleep problems” (OWM1:22).* Her mother also added, *“She seems to have difficulties with playing actively and does not like to tidy up after play time” (OWM2:33).*

**The girl said***, “I do not like my classmate XX; he often says to me that I should not sit on this seat” (OWC1:34). “I am afraid of my teacher… she will punish me because I work slowly” (OWC2:45).*

#### Case 3: The underweight boy

This boy is 5 11/12 years old with a BMI of 13.3, placing him in the underweight category. He shows impaired physical, social, and emotional functioning and shows poor school performance. He is the second child in the family. His older brother is twelve years old and looks after him after school until their parents come home. He is attending a full-day kindergarten with average school fees. Both parents have a primary education and work full time as a chef and a cashier. His parents immigrated to Hong Kong from Mainland China twenty years ago. The family lives in a private flat. Both the teacher and his mother have realised that the boy has difficulty performing well physically and in school, as follows:

**The teacher said,***“…. he certainly is not able to walk a street; he always has difficulties running about in school… but at times it seems he cannot keep still” (UT1:32). “He looks sad and worried… sometimes he cannot get along with other children, and he seems to have few friends at school. He often forgets things and loses his concentration during class” (UT2:59).*

**His mother said,***“… he appears to have difficulty tidying up toys after playing [for physical reasons]… He is often angry with people, and he seems to be unable to get along with other children” (UM1:45). “… he sometimes complains that he suffers from pain and bruises. He finds it difficult to concentrate on his homework, even if I sit beside him” (UM2:60).*

**The boy said,***“I always have abdominal pain and headache. That is why I am absent from school. I am often scared and have horrible dreams. I am afraid that the house will catch fire… Er…because my parents are never at home” (UC1:26) “…. My teacher does not like me because I cannot do the work properly…I only have one friend at school” (UC2:25).*

#### Case 4: The normal weight girl

This girl is 5 7/12 years old with a BMI of 14.6, a normal or healthy weight. She is doing well in terms of school performance, as well as in her social and emotional functioning. She is the second child in her family and her older sister is 16 years old. She attends a full-day kindergarten with low school fees. Both parents are originally from Hong Kong and have secondary education. They both have full-time work as a technician and a secretary. They live in a private flat. The girl is cared for by her grandmother. According to both her teacher and mother, the girl does well in her physical functioning, except sometimes she is not willing to tidy up toys after playing. Both her teacher and her mother thought that although she sometimes could not concentrate well, she is functioning well emotionally and socially.

**The teachers said**, *“… a wonderful and capable kid, she does well in school work, is an active player, and is a motivated learner” (NT1:23). “Everyone likes her, but she sometimes forgets things during class” (NT2:34).*

**Her mother said**, *“… she likes to offer to help to me… she carried a bag of rice (10 kg in weight) while shopping with me” (NM1:23)*. “*She is not always a happy child, though she can play with people and children” (NM2:55).*

## Discussion

The primary purpose of this study was to investigate the associations between quality of life and weight status in young Chinese children. The main finding of this study, based on quantitative and qualitative analyses, was that abnormal weight children such as obese, overweight or underweight children are more likely to suffer from impaired quality of life compared to normal weight children. Our quantitative findings are particularly supported by previous studies, which show that obese children generally suffer from greatly impaired physical health and social disadvantages [1,34], as well as difficulties in school performance compared to healthy weight children [35]. National Institute for Health Care Management (NIHCM) Foundation has stressed that obesity is associated with poorer academic performance beginning as early as kindergarten [36]. Conversely, normal-weight children have been found to have higher school functioning and social functioning scores than all three types of abnormal-weight children, and better physical health than underweight and obese children [37], findings that correspond to those of the current study.

One of the strengths of this study is that we used mixed methods, a new approach in this literature. This allowed us to draw conclusions based both on the mothers’ perceptions of their children’s quality of life from the quantitative data and the mothers’, teachers’ and the children’s comments from the case studies. The key limitation of many mixed-method studies is that there is a loss of information when qualitative data are quantified [38]. However, in the current study we retained the qualitative data in its original form and did not convert it for purposes of quantitative statistical analysis. Our findings from the quantitative and qualitative data were quite similar, with some exceptions. Quantitative analyses showed no statistically significant difference in the Physical Functioning scores of normal weight and overweight children, but the case study data provided examples of physical problems experienced by the overweight children, such as the Case 2 girl, whose mother and teacher comment: “… t*o have difficulties with playing actively”; “.. she is unable to play certain activities in the school…”*.

Moreover, the quantitative data showed that the abnormal-weight children encountered a certain degree of interruptions to their School Performance and Social Functioning but not to their Emotion Functioning. However, the findings from the case studies indicated that the obese children were deeply worried and unhappy. As one child stated, *“I am sad because my mother is feeling sad…”, “I am scared the house will catch fire because my parents are not at home…” and “I am afraid of my teacher dislikes me because I work slowly… and I am afraid there is a ghost because the flat is too dark…and the teacher punished me because I left the classroom without telling her.”* It is necessary to pursue further the children’s stories on the socio-emotional aspect of health when assessing young children’s health-related quality of life. Data from the case studies on the association of young children overweight or obesity with lower levels of emotional functioning may too spare to draw conclusion. Most importantly, future studies should investigate further the extent of causality between a young child’s weight and the contextual quality of life measures.

This study demonstrates that overweight and obesity of young children in Hong Kong are associated with the issue of multiple childcare arrangement arrangements. Based on the parent survey data in this study, 40% of primary caregivers were relatives or childcare providers; of the 10 case studies, only two mothers were full-time workers and the child was also cared for either by grandparents, in-house maids or older siblings. In this respect, the total sample was more representative of Hong Kong families in general than were the subset of families described in the case studies. According to the Hong Kong Government, there are approximately 273,609 foreign domestic helpers employed as childcare providers for young families in Hong Kong [39]. Reliance on multiple caregivers and on childcare outside the home is especially evident among more affluent “Westernised” and educated parents who are likely both to be working full time [40]. In the current study, the low quality of life experienced by some children may be attributable in part to disruptions associated with childcare arrangements, parenting difficulties, or/and special familial circumstances.

Increasing evidence shows that paediatric obesity is linked to family socio-demographics. Our study in Hong Kong showed that obese children come from situations of social disadvantage, with poor housing and newly immigrated families with cross-boundary marriages (e.g., bridegrooms are Hong Kong residents and brides are from mainland China) [41]; the majority of these families reported receiving social welfare benefits [42]. This is to support previous studies that obese children generally suffer from physical and social disadvantages associated with poor quality of life [43,44], and obesity has also been framed as a consequence of poor lifestyle choices and increased consumption of low-cost, energy-dense/nutrient-poor foods. Jenkin et al. [9] stressed that obesity policies must address broader determinants of health, such as social inequalities.

However, more recent research findings reveal that the relationship between socio-economic characteristics and obesity in children may not follow consistent trends according to age and gender or over time [45,46] because QoL is context specific, and what impacts one group of children's QoL within a particular context may not impact another group in a different situation [47]. A large heterogeneous sample should be recruited to investigate which food-intake behaviours or child food consumption practices contributing to excess weight gain are specific to or more prevalent in certain SES groups. Future studies should also investigate the relative importance of socio-cultural attitudes and beliefs of parenting regarding obesity versus the physical, psycho-social, economic, and policy environments of children and their families.

One of the limitations of this study is that the findings cannot be generalised beyond the Hong Kong setting. Furthermore, this sample was based on voluntary preschool community participation, and because we did not recruit in clinical settings, the results likely under represent the experiences of abnormal-weight children who have an even poorer quality of life than the study sample. Nevertheless, the present study reveals similar findings to those of the Hong Kong government’s needs assessment of children under five years of age in Hong Kong [48], which demonstrated that the majority of children in this age group are given insufficient attention by their parents when both parents work. These parents spend a small amount of time with their children, who generally experience considerable stress. Furthermore, in families from low socio-economic backgrounds, single-parent families and new immigrant families, parents experience more problems in parenting.

This special obesity-link phenomenon is supported by Garasky et al. [49], who note positive associations between a range of family stressors and children’s problems with being overweight or obese, such as lack of cognitive stimulation and emotional support in the household among younger children and financial strain in households among older children. One issue that should be considered is how public policies that reduce family stressors may, in turn, help reduce childhood obesity because of an improved quality of life.

## Conclusion

This study is one of only a few studies that have examined parents’, teachers’ and the young children’s own perceptions of the children’s quality of life across different weight statuses. As such, the findings of the current study may contribute to the understanding of most of quantitative findings, through which may develop obesity interventions, and the development of new theoretical models, for greater utility in childhood obesity research. This mixed method study has supplemented the extent literature by providing a better understanding of the relationship between young children’s quality of life and child weight under a rapid socio-economical changing zone, which may encourage kindergartens, families, community organisations and healthcare providers not only to promote changes in physical activity and food environments but also to foster an “eco-behavioural-environmental” synergy.

## Competing interests

The authors declare that they have no competing interests.

## Authors’ contributions

C MS C and W-C W carried out the “Quality of life in overweight and obese young Chinese children: A mixed–method study” studies, participated in the sequence alignment and drafted the manuscript. All authors read and approved the final manuscript. The authors declare that they have no competing interests.

## Supplementary Material

Additional file 1 Pediatric Quality of Life Inventory. Click here for file

Additional file 2 Demographic Characteristics of Children and Parents by Weight Group (n = 336). Click here for file
