# Supplementary material for: Quality of life in overweight and obese young Chinese children: a mixed-method study
Source: Health Qual Life Outcomes. 2013 Mar 6;11:33. doi: 10.1186/1477-7525-11-33 (PMC3605313; doi:10.1186/1477-7525-11-33)
Supplement: Additional file 1 — Pediatric Quality of Life Inventory. [file 1477-7525-11-33-S1.pdf]

ID#
____
Date:
____

PedsQL™

Pediatric Quality of Life Inventory

Version 4.0

YOUNG CHILD REPORT (ages **5-7**)

Instructions for interviewer:
I am going to ask you some questions about things that might be a problem for some children. I want to know how much of a problem any of these things might be for you.

Show the child the template and point to the responses as you read.

If it is not at all a problem for you, point to the smiling face
If it is sometimes a problem for you, point to the middle face
If it is a problem for you a lot, point to the frowning face

I will read each question. Point to the pictures to show me how much of a problem it is for you. Let's try a practice one first.

	**Not at all**	**Sometimes**	**A lot**
**Is it hard for you to snap your fingers**	☺	😐	☹

Ask the child to demonstrate snapping his or her fingers to determine whether or not the question was answered correctly. Repeat the question if the child demonstrates a response that is different from his or her action.

PedsQL 4.0 – (5-7) Not to be reproduced without permission Copyright© 1998 JW Varni, Ph.D. All rights reserved

Review copy

Think about how you have been doing for the last few weeks. Please listen carefully to each sentence and tell me how much of a problem this is for you.

After reading the item, gesture to the template. If the child hesitates or does not seem to understand how to answer, read the response options while pointing at the faces.

**PHYSICAL FUNCTIONING** ***(problems with…)***	**Not at all**	**Some-times**	**A lot**
1. Is it hard for you to walk	0	2	4
2. Is it hard for you to run	0	2	4
3. Is it hard for you to play sports or exercise	0	2	4
4. Is it hard for you to pick up big things	0	2	4
5. Is it hard for you to take a bath or shower	0	2	4
6. Is it hard for you to do chores (like pick up your toys)	0	2	4
7. Do you have hurts or aches (***Where? ____________***)	0	2	4
8. Do you ever feel too tired to play	0	2	4

Remember, tell me how much of a problem this has been for you for the last few weeks.

**EMOTIONAL FUNCTIONING** ***(problems with…)***	**Not at all**	**Some-times**	**A lot**
1. Do you feel scared	0	2	4
2. Do you feel sad	0	2	4
3. Do you feel mad	0	2	4
4. Do you have trouble sleeping	0	2	4
5. Do you worry about what will happen to you	0	2	4

**SOCIAL FUNCTIONING** ***(problems with…)***	**Not at all**	**Some-times**	**A lot**
1. Is it hard for you to get along with other kids	0	2	4
2. Do other kids say they do not want to play with you	0	2	4
3. Do other kids tease you	0	2	4
4. Can other kinds do things that you cannot do	0	2	4
5. Is it hard for you to keep up when you play with other kids	0	2	4

**SCHOOL FUNCTIONING** ***(problems with…)***	**Not at all**	**Some-times**	**A lot**
1. Is it hard for you to pay attention in school	0	2	4
2. Do you forget things	0	2	4
3. Is it hard to keep up with schoolwork	0	2	4
4. Do you miss school because of not feeling good	0	2	4
5. Do you miss school because you have to go to the doctor’s or hospital	0	2	4

PedsQL 4.0 – (5-7) Not to be reproduced without permission Copyright© 1998 JW Varni, Ph.D. All rights reserved

Review copy

How much of a problem is this for you?

Not at all	Sometimes	A lot
☺	😐	☹

PedsQL 4.0 – (5-7) Not to be reproduced without permission Copyright© 1998 JW Varni, Ph.D. All rights reserved

Review copy

ID# ___________________________

Date: ___________________________

PedsQL™

Pediatric Quality of Life Inventory

Version 4.0

PARENT REPORT for **YOUNG CHILDREN** (ages **5-7**)

DIRECTIONS

On the following page is a list of things that might be a problem for **your child**. Please tell us **how much of a problem** each one has been for **your child** during the **past ONE month** by circling:

0 if it is **never** a problem
1 if it is **almost never** a problem
2 if it is **sometimes** a problem
3 if it is **often** a problem
4 if it is **almost always** a problem

There are no right or wrong answers.
If you do not understand a question, please ask for help.

PedsQL 4.0 – Parent (5-7) Not to be reproduced without permission Copyright© 1998 JW Varni, Ph.D. All rights reserved

Review copy

*In the past **ONE month,** how much of a **problem** has your child had with…*

**PHYSICAL FUNCTIONING** ***(problems with…)***	**Never**	**Almost Never**	**Some-times**	**Often**	**Almost Always**
1. Walking more than one block	0	1	2	3	4
2. Running	0	1	2	3	4
3. Participating in sports activity or exercise	0	1	2	3	4
5. Taking a bath or shower by him or herself	0	1	2	3	4
6. Doing chores, like picking up his or her toys	0	1	2	3	4
7. Having hurts or aches	0	1	2	3	4
8. Low energy level	0	1	2	3	4

**EMOTIONAL FUNCTIONING** ***(problems with…)***	**Never**	**Almost Never**	**Some-times**	**Often**	**Almost Always**
1. Feeling afraid or scared	0	1	2	3	4
2. Feeling sad or blue	0	1	2	3	4
3. Feeling angry	0	1	2	3	4
4. Trouble sleeping	0	1	2	3	4
5. Worrying about what will happen to him or her	0	1	2	3	4

**SOCIAL FUNCTIONING** ***(problems with…)***	**Never**	**Almost Never**	**Some-times**	**Often**	**Almost Always**
1. Getting along with other children	0	1	2	3	4
2. Other kids not wanting to be his or her friend	0	1	2	3	4
3. Getting teased by other children	0	1	2	3	4
4. Not able to do things that other children his or her age can do	0	1	2	3	4
5. Keeping up when playing with other children	0	1	2	3	4

**SCHOOL FUNCTIONING** ***(problems with…)***	**Never**	**Almost Never**	**Some-times**	**Often**	**Almost Always**
1. Paying attention in class	0	1	2	3	4
2. Forgetting things	0	1	2	3	4
3. Keeping up with school activities	0	1	2	3	4
4. Missing school because of not feeling well	0	1	2	3	4
5. Missing school to go to the doctor or hospital	0	1	2	3	4

PedsQL 4.0 – Parent (5-7) Not to be reproduced without permission Copyright© 1998 JW Varni, Ph.D. All rights reserved

Review copy

ID#

____

Date:

____

PedsQL™

Pediatric Quality of Life Inventory

Version 4.0

PARENT REPORT for **TODDLERS** (ages **2-4**)

DIRECTIONS

On the following page is a list of things that might be a problem for **your child**. Please tell us **how much of a problem** each one has been for **your child** during the **past ONE month** by circling:

0 if it is **never** a problem
1 if it is **almost never** a problem
2 if it is **sometimes** a problem
3 if it is **often** a problem
4 if it is **almost always** a problem

There are no right or wrong answers.
If you do not understand a question, please ask for help.

PedsQL 4.0 – Parent (2-4) Not to be reproduced without permission Copyright© 1998 JW Varni, Ph.D. All rights reserved

Review copy

*In the past **ONE month,** how much of a **problem** has your child had with…*

**PHYSICAL FUNCTIONING** ***(problems with…)***	**Never**	**Almost Never**	**Some-times**	**Often**	**Almost Always**
1. Walking	0	1	2	3	4
2. Running	0	1	2	3	4
3. Participating in active play or exercise	0	1	2	3	4
4. Lifting something heavy	0	1	2	3	4
5. Bathing	0	1	2	3	4
6. Helping to pick up his or her toys	0	1	2	3	4
7. Having hurts or aches	0	1	2	3	4
8. Low energy level	0	1	2	3	4

**EMOTIONAL FUNCTIONING** ***(problems with…)***	**Never**	**Almost Never**	**Some-times**	**Often**	**Almost Always**
1. Feeling afraid or scared	0	1	2	3	4
2. Feeling sad or blue	0	1	2	3	4
3. Feeling angry	0	1	2	3	4
4. Trouble sleeping	0	1	2	3	4
5. Worrying	0	1	2	3	4

**SOCIAL FUNCTIONING** ***(problems with…)***	**Never**	**Almost Never**	**Some-times**	**Often**	**Almost Always**
1. Playing with other children	0	1	2	3	4
2. Other kids not wanting to play with him or her	0	1	2	3	4
3. Getting teased by other children	0	1	2	3	4
4. Not able to do things that other children his or her age can do	0	1	2	3	4
5. Keeping up when playing with other children	0	1	2	3	4

****Please complete this section if your child attend school or daycare***

**SCHOOL FUNCTIONING** ***(problems with…)***	**Never**	**Almost Never**	**Some-times**	**Often**	**Almost Always**
1. Doing the same school activities as peers	0	1	2	3	4
2. Missing school/daycare because of not feeling well	0	1	2	3	4
3. Missing school/daycare to go to the doctor or hospital	0	1	2	3	4

PedsQL 4.0 – Parent (2-4) Not to be reproduced without permission Copyright© 1998 JW Varni, Ph.D. All rights reserved

Review copy
