# Supplementary material for: Quality of life in overweight and obese young Chinese children: a mixed-method study
Source: Health Qual Life Outcomes. 2013 Mar 6;11:33. doi: 10.1186/1477-7525-11-33 (PMC3605313; doi:10.1186/1477-7525-11-33)
Supplement: Additional file 2 — Demographic Characteristics of Children and Parents by Weight Group (n = 336). [file 1477-7525-11-33-S2.doc]

**Additional File 1.** Demographic Characteristics of Children and Parents by Weight Group (n = 336).

| Demographic Characteristics | | | | Underweight  *(BMI: 11.3-14.7)* (n=94) | | Normal weight  *(BMI: 14.1-17.5)*  (n=94) | Overweight  *(BMI: 17.2-19.5)*  (n=93) | Obese  *(BMI: 19.2-28.0)*  (n=55) | *p* value |
| --- | --- | --- | --- | --- | --- | --- | --- | --- | --- |
|  | | | | % | | % | % | % |  |
| Children | | |  |  | | |  |  |  |
| Gender | | |  |  | | |  |  | .707 |
|  | Girl (n=156) | |  | 30.1% | | 26.3% | 28.8% | 14.7% |  |
|  | Boy (n=180) | |  | 26.1% | | 29.4% | 26.7% | 17.8% |  |
| Age range | | |  |  | |  |  |  | .000 |
|  | 2-4 (n=176) | | | 25.0% | | 38.6% | 21.6% | 14.8% |  |
|  | 5-7 (n=160) | | | 31.3% | | 16.3% | 34.4% | 18.1% |  |
| Number of siblings | | | |  | |  |  |  | .837 |
|  | 1 (n=202) | | | 27.7% | | 26.7% | 30.2% | 15.3% |  |
|  | 2 (n=105) | | | 29.5% | | 30.5% | 21.9% | 18.1% |  |
|  | 3 or more (n=29) | | | 24.1% | | 27.6% | 31.0% | 17.2% |  |
| Type of schooling | | |  |  | |  |  |  | .001 |
|  | Half day (n=159) | | | 30.2% | | 17.6% | 31.4% | 20.8% |  |
|  | Full day (n=177) | | | 26.0% | | 37.3% | 24.3% | 12.4% |  |
| Number of stages of childcare arrangements since birth | | | | | | |  |  | .552 |
|  | 1 (n=246) | | | 30.9% | | 27.2% | 26.4% | 15.4% |  |
|  | 2 (n=66) | | | 18.2% | | 28.8% | 33.3% | 19.7% |  |
|  | 3 or more (n=24) | | | 25.0% | | 33.3% | 25.0% | 16.7% |  |
| Type of housing | | | |  | |  |  |  | .000 |
|  | | Government rental housing (n=94) | | 24.5% | | 25.5% | 25.5% | 24.5% |  |
|  | | Private rental housing (n=60) | | 31.7% | | 38.3% | 25.0% | 5.0% |  |
|  | | Government-subsidised housing (n=66) | | 25.8% | | 43.9% | 15.2% | 15.2% |  |
|  | | Privately purchased housing (n=116) | | 30.2% | | 15.5% | 37.9% | 16.4% |  |
| Respondent | | | | |  | |  |  | .388 |
|  | Mother (n=303) | | | 27.4% | | 29.4% | 27.1% | 16.2% |  |
|  | Father (n=33) | | | 33.3% | | 15.2% | 33.3% | 18.2% |  |
| Perceived as principal caregiver | | | | |  | |  |  | .843 |
|  | Mother (n=191) | | | 30.4% | | 27.2% | 25.7% | 16.8% |  |
|  | Father (n=7) | | | 28.6% | | 14.3% | 42.9% | 14.3% |  |
|  | Other (relatives, child minders) (n=138) | | | 24.6% | | 29.7% | 29.7% | 15.9% |  |
| Mother | | | |  | |  |  |  |  |
| Mother’s birth place | | | | | |  |  |  | .321 |
|  | | China, Mainland (n=217) | | 28.1% | | 25.8% | 26.7% | 19.4% |  |
|  | | Hong Kong (n=111) | | 27.9% | | 33.3% | 28.8% | 9.9% |  |
|  | | Other (n=8) | | 25.0% | | 12.5% | 37.5% | 25.0% |  |
| Length of stay in Hong Kong | | | | | |  |  |  | .735 |
|  | | 5 years or less (n=100) | | 24.0% | | 27.0% | 29.0% | 20.0% |  |
|  | | 6 - 30 years (n=134) | | 31.3% | | 26.1% | 26.1% | 16.4% |  |
|  | | Over 30 years (n=102) | | 27.5% | | 31.4% | 28.4% | 12.7% |  |
| Mother’s education level | | | | | |  |  |  | .356 |
|  | | Primary (n=51) | | 29.4% | | 27.5% | 31.4% | 11.8% |  |
|  | | Secondary (n=248) | | 27.0% | | 26.6% | 27.4% | 19.0% |  |
|  | | Tertiary (n=37) | | 32.4% | | 37.8% | 24.3% | 5.4% |  |
| Mother’s employment status | | | | | |  |  |  | .569 |
|  | | Unemployed /housewife (n=150) | | 26.0% | | 30.7% | 28.0% | 15.3% |  |
|  | | Part-time (n=30) | | 36.7% | | 20.0% | 23.3% | 20.0% |  |
|  | | Full-time (n=99) | | 28.3% | | 34.3% | 27.3% | 10.1% |  |
| Father | | | |  | |  |  |  |  |
| Father’s birth place | | | |  | |  |  |  | .649 |
|  | | China, Mainland (n=145) | | 27.6% | | 24.1% | 30.3% | 17.9% |  |
|  | | Hong Kong (n=184) | | 28.3% | | 31.0% | 25.0% | 15.8% |  |
|  | | Other (n=7) | | 28.6% | | 28.6% | 42.9% | 0.0% |  |
| Length of stay in Hong Kong | | | | | |  |  |  | .779 |
|  | | 5 years or less (n=30) | | 30.0% | | 33.3% | 26.7% | 10.0% |  |
|  | | 6 - 30 years (n=116) | | 25.9% | | 27.6% | 31.9% | 14.7% |  |
|  | | Over 30 years (n=190) | | 28.9% | | 27.4% | 25.3% | 18.4% |  |
| Father’s education level | | | | | |  |  |  | .122 |
|  | | Primary (n=47) | | 25.5% | | 17.0% | 42.6% | 14.9% |  |
|  | | Secondary (n=228) | | 29.8% | | 27.6% | 25.0% | 17.5% |  |
|  | | Tertiary (n=61) | | 23.0% | | 37.7% | 26.2% | 13.1% |  |
| Father’s employment status | | | | | |  |  |  | .509 |
|  | | Unemployed (n=5) | | 0.0% | | 60.0% | 20.0% | 20.0% |  |
|  | | Part-time (n=12) | | 16.7% | | 25.0% | 41.7% | 16.7% |  |
|  | | Full-time (n=271) | | 30.6% | | 29.2% | 25.5% | 14.8% |  |

*Note*: The stage of childcare arrangements was defined by the child having been cared for by different primary caregivers and/or at different places.

The BMI values for weight categories overlapped because according to the IOTF definition of weight status, there are different BMI ranges based on sex and age. As a result, there is overlapping of BMIs for different weight status categories.

The child weight status was defined by the IOTF (1985).

*p* value based on Chi-square test and Fisher’s exact test.
